# Epidemic Cholera—Its History, Causes, Pathology and Treatment

**Published:** 1849-07

**Authors:** 


### Part 2.—Reviews and Notices of New Works.

### ARTICLE I.

*Epidemic Cholera: its History, Causes, Pathology and Treatment.* By C. B. Coventry, M. D., Professor of Obstetrics and Medical Jurisprudence in the Medical Institution of Geneva College; Professor of Physiology and Medical Jurisprudence in the University of Buffalo. "Internal sanatory arrangements, and not quarantine or sanitory lines, are the safeguards of nature against epidemic diseases."---*Farr.---Registrar General.* Buffalo: Geo. H. Derby & Co.: 1849. From Publishers.

The small volume with the above title, giving, as it does, a condensed history of epidemic cholera, together with a synopsis of the various theoretical views with regard to its cause, and opinions as to the best treatment for the different stages, will prove a valuable source of information to such as may wish to consult authorities upon this subject.

The remarks of the author upon the causes, pathology, and treatment of the disease, are preceded by a brief history of its appearance and progress, at different times and in various countries, under various forms and degrees; in some instances manifesting itself in a mild form and attended with even less mortality than many other eipdemics; and at other times and in other places, casting before it the shadows of fear and doubt, and scattering in its path devastation and ruin. "Somerset, who traveled the Coromandel coast in 1774 and 1781, speaks of a disease resembling cholera, prevailing as an epidemic, and states that in one visitation of the disease, above 60,000 people, from Cherigan to Pondicherry, perished." "The Report of the French Commissioners says it first appeared in Bombay on the 11th of August, 1820, and carried off previous to the month of February following, eleven hundred and thirty victims. Again it returned in September of 1821; accompanied by excessive heat, and destroyed from the 23d to the 28th, two hundred and thirty-five persons." "The cholera made its appearance at Sunderland, in England, in October, 1831. On the 28th of November, there had been two hundred and ninety-four cases, of which sixty-eight had proved fatal, and thirty-two remained under treatment at the time of the report. It soon after appeared in London, and on the 18th of April, there had been twenty-four hundred and seventy-seven cases, of which twelve hundred and one were fatal. In Dublin, ninety-four cases and fifty-eight deaths. In Cork, eighty-five cases and thirty-eight deaths."

The history of the disease at all times and in all countries, tends to prove that, as is the case with most other epidemics, hot, damp weather, low, marshy districts, and narrow, damp, and filthy streets, determine in a great measure the time and place of its appearance and the degree of its malignity.

In the chapter upon the causes of the disease the author takes strong grounds against those who believe in its contagiousness. "When we remembered," says he, "the panic which prevailed in 1832—the vexation caused and the money expended in useless quarantines—the hurried and barbarous interment of the dead, scarce waiting till the breath had left the body—the worse than brutal desertion and neglect of friends and relations—all growing out of a belief in the contagious nature of the disease; we rejoiced in the belief that, whatever of suffering a superintending Providence might see fit to inflict, we would not again thus aggravate them by our own acts." * * * "If the decisive and explicit testimony of Annesley, who, for five years, had charge of a hospital where cholera patients were mingled indiscriminately with those from other diseases, and who tells us that not more than six or seven cases originated in the hospital during that time; the no less emphatic testimony of Dr. Searle, who had the disease in India, and who informed us, that when in charge of the hospital at Warsaw, but two cases originated in the Hospital, and that he himself slept in the bed in which a gentleman had died of the disease the preceding night without his contracting it---if the testimony of Annesley and others in India, that when the disease attacked a corps of the army, it was soonest got rid of by separating them into small detachments---if the fact, which was notorious in Asia, Europe, and America, that when the disease attacked large cities the population, in fleeing into the country and neighboring villages, did not carry the disease with them---we repeat, if these facts, together with the experience of the profession from Calcutta to Moscow, and from Moscow to Quebec, and from Quebec to New Orleans, is not sufficient to settle the question of contagion, we are at a loss to see how it ever can be settled."

Almost uniformly it passes from east to west, following the course of large streams and great thoroughfares, and making its appearance suddenly and unexpectedly in one place immeately after abating in another.

In some instances it has been known to disappear from a locality suddenly, and return again after a time with as much violence as before. A single town or village has often suffered from its ravages, whilst the country and towns even in the vicinity have been entirely exempt. It is, therefore, impossible to draw any very definite conclusions as to its cause from the history of its rise and progress.

The causes may be divided, as the author remarks, into, "1st, those that affect the whole community of a place, and, 2d, those that affect the individuals. First, sporadic causes, affecting the whole community of a city or village, are what is usually termed malaric, or, in other words, bad air. This arises, in a great measure, from the decomposition of animal and vegetable matter. It is often found in its greatest intensity, in low, damp, and marshy situations, in the neigborhood of stagnant pools of water—it is always generated in crowded cities, and where large numbers of persons are crowded together, as in armies. It is increased in cities, by close, narrow streets, preventing proper ventilation—by accumulation of filth in the streets, in dwellings, or about the premises; in short, whereever there are accumulations of vegetable and animal matter there is a focus for the generation of malaria."

In addition to the local causes, there are others affecting individuls, such as exhaustion from over exertion, debility consequent upon previous disease, want of sufficient nourishment or clothing, and excesses in eating or drinking.

To these may be added another, perhaps the most potent, cause. We refer to the depressing influence of fear. On this subject the author makes the following, in our opinion, very appropriate and just remarks.

It is well known that there is no moral influence which produces so depressing an effect on the system as fear. It has been clearly proved that the most vigorous of men, even in the most perfect health, may be frightened to death. We have known nervous and irritable persons, who were always thrown into a diarrhœa when much alarmed. If we only look at a frightened person, we see that they present almost the first symptoms of cholera—the face is pale, the surface cold, the pulse feeble, the blood having retreated from the surface to the central organs of the body. Could we satisfy community of what we fully believe, that the epidemic cause is seldom or never sufficient to produce the disease, and that they have nothing to fear so long as they avoid the other and local causes, we should confer the greatest possible benefit on the public.

The symptoms of cholera have been so often and graphically described, that it seems unnecessary to occupy time and space in a recapitulation of signs and indications so peculiar and well marked as those indicative of this disease.

It may be briefly stated that there are properly three stages: the incipient, or first, attended with lassitude, slight pain in the abdomen, nausea and moderate diarrhœa, or either of the above symptoms may be considered premonitory whenever they occur during the prevalence of the epidemic. At least it is safe at such times to look upon them in that light so that remedies may be used promptly. In the second stage, all the characteristic symptoms of cholera are most generally present, such as oppressed breathing, chilliness, coldness of extremities, weak and instable pulse, cramps, general or partial, vomiting and purging of a thin fluid substance in large quantites.

Cases may, and frequently do, occur in which but two or three of these characteristic symptoms are present.

The third stage which uniformly follows the second, if speedy relief is not obtained from the above named symptoms, is indicated by a sunken, almost death-like appearance of the countenance, ice-like coldness of the surface, intermittent pulse in the large vessels, it being frequently entirely absent at the wrists, and a condition of the hands which is peculiar and cannot be mistaken, appearing, as they do, as if they had been soaked for a long time in water.

Our author contends that the first is the only stage of the disease that can properly be said to be curable, hence the importance of using efficient remedies at the very onset.

The great mistake, as he contends, is to consider the first symptoms as premonitory merely, when they are indicative of this first stage requiring prompt and efficient treatment.

The views of the author upon the treatment of the different stages of the disease are embraced in the following quotations from the chapter upon the subject.

In the first stage of the disease, the patient should confine himself to his bed and take some mild aromatic drink, such as infusion of spear-mint, or camomile, or warm camphor julep until reaction takes place. The perspiration which follows should be encouraged by diluent drinks: this may also be promoted by a powder composed of three or four grains of Dover's powder and one of calomel; or a pill composed of camphor, gr. ss, opium, gr. one-fourth, calomel, gr. i, repeated every two hours; after the sweating has continued three or four hours, the surface should be dried with warm flannel cloths, and a fresh and clean dress of flannel put on. When five or six ot the pills have been taken, they should be suspended, and a small dose of rhubarb and magnesia, or of pnre castor oil should be given. Active, or drastic, or saline cathartics should not be given, and if castor oil is used, great care should be taken to see that it is pure and fresh. Most disastrous consequences have resulted from giving castor oil that was rancid. Mild nourishment should be given from time to time. If found necessary, the pills and cathartics may be repeated. The object should be to remove nervous prostration, and congestion which has already commenced, by equalizing the circulation.

*Treatment of the second Stage.* If the patient was strong and plethoric, the pulse still full and distinct, the cramp severe, or there was great oppression in breathing, we would put the feet and legs in water as warm as could be borne, with the addition of mustard and common salt to the water; open a vein in the arm, and bleed from five to sixteen or twenty ounces, watching the effect produced on the pulse and system generally; then place the patient in a warm bed, apply warmth to the feet and along the limbs; apply a large mustard cataplasm over the stomach, and give one of the pills of calomel, opium, and camphor, every half hour. If the desire for cold drinks is not strong, the patient may drink from time to time a weak infusion of spearmint, with the addition of eight or ten drops of camphorated spirits; if however, the thirst is very intense, cold water may be used instead of the tea, and, in addition small bits of ice given from time to time. The bed should be placed in the centre of the room, without curtains, and the room should, if possible, be large, well ventilated, and if an upper room the better. If the weather will admit, the doors and windows should be kept open---the best stimulus for the patient is plenty of pure air---if the weather is damp, or chilly, so as to render it necessary to close the windows and doors, a little fire should be placed in an open fireplace, to temper the air; no persons should be admitted into the room except such as are necessary; every additional person renders the air more impure. The warm applications should be changed from time to time. If the patient be much exhausted or vomiting, on no consideration should he be permitted to use the night vessel---the effort o getting up revives the vomiting, and this in turn brings on the alvine discharges. If the patient be feeble and delicate, or if the discharges have been profuse, or if the fluids of the system have been drained off by long continuance of diarrhœa in the first stage, the bleeding must be omitted, and the other means adopted as before. If there is great precordial oppression, cups may be applied over the region of the stomach, and the mustard over the abdomen and over the stomach after the removal of the cups. If the pulse becomes gradually more full and distinct, and warmth returns to the surface, we have only to persevere in these means; after a time giving the pills less frequently, and permitting the patient to take some light nourishment as chicken tea or light broth. If the perspiration becomes profuse---as will be likely---with a warm skin, it should be encouraged by taking some tepid aromatic drinks; the spearmint tea with a few drops of camphor is as good as anything. If, notwithstanding these means the patient continues to sink, or the profuseness of the discharges continues, we must resort to other means to try and arouse the system to reaction. Much care is, however, necessary to avoid throwing the patient into the fourth stage. Sulphuric ether in small doses should be given, and repeated every ten or fifteen minutes; or a weak solution of carb. of ammonia may be substituted. At the same time an enema of a pint of chicken tea, with a table-spoonful of table-salt, should from time to time be thrown into the bowels, and its retention secured for a few minutes by pressure on the fundament; a sheet should be placed under the patient to receive the discharges, and on no account should he be permitted to rise from the bed. If the symptoms of reaction come on, the use of the stimulants should be gradually suspended. We believe that the more permanent and powerful stimulants such as brandy, ardent spirits, etc., are only admissible where the patient has been addicted to their use. Too much precaution cannot be used during the period of convalescence as regards diet, exposure, etc., in all cases in which the disease had approached the stage of collapse.

